# Assessing fidelity: balancing methodology and reality in jail interventions

**DOI:** 10.1186/s12905-018-0617-x

**Published:** 2018-07-23

**Authors:** Patricia J. Kelly, Amanda Emerson, Chelsea Fair, Megha Ramaswamy

**Affiliations:** 1 School of Nursing and Health Studies, University of Missouri-Kansas City, 2464 Charlotte St, Kansas City, MO 64108 USA; 2 Department of Family Medicine, University of Kansas Medicine Center, Kansas City, KS USA; 3 Department of Preventive Medicine, University of Kansas Medical Center, Kansas City, KS USA

**Keywords:** Criminal justice, Community health, Structural barriers, Research methods

## Abstract

**Background:**

While fidelity to research protocols is important to ensure generalizable outcomes, interventions in criminal justice settings present unique challenges to uniform implementation. The goal of this paper is to describe the fidelity methods and outcomes for a sexual health intervention implemented in three local county jails.

**Methods:**

As part of a longitudinal cohort study, four trained fidelity assessors observed 25 of the 230 sessions presented (including both intervention and comparison groups) at three separate times during the 29 months of the intervention. Assessment methods included the assessors’ field notes, a nine-item facilitator quality scale and a content inclusion scale with 6–13 items specific for each of the five sessions.

**Results:**

Facilitator quality score ranged from 87.6 to 99.2%. Content inclusion scores ranged from 77.3 to 88%. Specific challenges to fidelity were found in two areas: the jail environment and the participants’ response to content.

**Conclusions:**

The realities of conducting research in jails and prisons must be addressed in real time by adjusting program content to fit both unexpected facility and participant situations. Skilled facilitators are essential to this effort.

## Background

“Frequently discussed, seldom evaluated” is an apt description of intervention fidelity in criminal justice settings. Frequently discussed because researchers are aware of the necessity of adhering to a protocol that delivers the same content over multiple sessions in order to accurately assess the impact of an intervention. Seldom evaluated because few interventions implemented in jails and prisons, even those delivered over multiple sessions or to multiple groups, develop a formal mechanism for the evaluation of their fidelity to the protocol. Fidelity, or the assurance of consistency to an intervention protocol, is important to maintain the integrity of any program. Fidelity is also important for both internal and external validity, assuring that program outcomes can be attributed to intervention participation [1].

Interventions in criminal justice settings seek to provide programming content that is not generally available for high-risk populations, and often focus on substance abuse, mental health disorders, and infectious diseases such as HIV infection, TB, and hepatitis C. Health issues specific to the smaller but more vulnerable population of incarcerated women have included contraceptive access, STI screening, and trauma-informed treatment [2, 3]. However, all interventions in these settings present unique challenges to protocol fidelity. Scheduled programs are often canceled due to facility lockdowns, sessions can begin late or end early because of staffing issues, participants may be absent as a result of disciplinary actions, and correctional officers’ differing desires for proximity demand flexibility for researchers implementing correctional interventions. Because researchers’ desires for education, programming and evaluation are different from corrections’ emphasis on safety and security, conflicts can arise if communication and tolerance are not maintained. Understanding how these differences impact program fidelity can assist researchers in developing strategies that take into account the inherent realities of implementing interventions in correctional setting, while also adhering to research protocols.

The goal of this paper is to describe the fidelity methods and outcomes for a sexual health intervention implemented in three local county jails. Results can be used to ensure the causal link between program participation and outcomes.

## Methods

The intervention of the parent study used the quasi-experimental design of a wait-listed comparison group method, in which all participants for each program were recruited on day one and randomly assigned to two groups; one group of which received the intervention in week one and the second wait-listed group which received the intervention in week two. Randomization occurred according to seating of consented participants: every other person went to group A (intervention group), the rest went to group B (wait-listed control group).

We assessed the fidelity of the intervention using the model of Wilson and colleagues [4]. The assessments occurred after 50, 100, and 150 women were recruited into the intervention, for a total of 25 of the 230 intervention and wait-listed control group sessions.

### Sample/site

Participant recruitment occurred at three county jails in the Kansas City metro area. The two urban jails had capacity for 800 and 300 inmates each; the third jail in a suburban location had capacity for 1000 inmates. Fifteen percent of the total population was female.

A total of 238 female participants were recruited for enrollment on a rolling basis at minimum and medium security housing units in 26 intervention cohort groups across the three facilities between September 2014 and March 2016. Women were recruited into the study using flyers and word of mouth from jail staff. Interviewers read a standardized recruitment script and consent form to each potential participant and administered a face-to-face survey in a semi-private space at the jail for those providing written consent. As compensation for participating, each woman was given a $5 credit to her commissary account or a gift basket worth $5.

### Intervention

The SHE Project was a cervical health literacy intervention designed to improve incarcerated women's knowledge about cervical health, reduce barriers to screening and treatment that stem from beliefs about cervical cancer, improve self-efficacy for cervical cancer screening and follow-up, and increase women’s confidence for navigating interactions with health care providers and systems [5]. The content of these individual sessions was driven by previous work on the cervical health literacy of incarcerated women, and the general literature on cancer health literacy, trauma-informed care and barriers to care and follow-up faced by incarcerated women [6–11]. Content delivery was rooted in social and feminist theory [12, 13]. We sought to understand women’s experiences within their social and political contexts, emphasizing: the role of romantic and sexual partnerships, family, community and trauma in women’s lives; the impact of race, class, and gender on specific health outcomes; and a rejection of status quo values and assumptions about women in general.

The intervention was delivered in a small group format and consisted of five, two-hour sessions, starting on a Monday and ending on a Friday. The themes of the sessions were: Knowledge about Sexual Health, Beliefs, Self-Efficacy, Navigation of Health Care Systems and Providers, and Wrap-Up/Summary. Session 1 included the completion of consent forms and baseline surveys, and Session 5 included the completion of a post-test and participant satisfaction survey, literature for participants to keep as a review, and distribution of a graduation certificate [5].

### Data collection

Fidelity evaluation data was collected by four separate fidelity assessors who had attended at least five staff meetings and were trained by program staff on all aspects of the intervention, including background about incarcerated populations, study protocol, and the implementation methods. Each fidelity assessor attended at least two intervention sessions prior to the formal assessment to assure familiarity with the setting, the intervention, and the population. The fidelity assessors observed two intervention sessions in the first week that the intervention was delivered (sessions 3 and 4) and returned the following week to observe sessions 1, 2 and 5 during the second week’s delivery of the intervention.

### Instruments

A facilitator quality score sheet was developed with seven items specific to the intervention (e.g., “encouraged open-ended discussion”, “expressed empathy”); scoring for this instrument was on a scale of 1 to 10, with 1 representing the lowest and 10 the highest quality rating. A second quantitative checklist of key intervention content components was developed, with 6–13 items specific for each of the five sessions; scoring was dichotomous (present/not present). Because only one assessor was present per session, inter-rater reliability was not an issue.

Each fidelity assessor wrote extensive field notes about their observations during the intervention sessions. Instructions for observation and field note writing included consideration of three questions:

- What unique challenges did the evaluator observe related to the jail setting?
- What strategies did the facilitator use to build rapport with participants?
- How did the participants relate to each other during the sessions?

Assessors were encouraged to take as many observational notes as possible relevant to their evaluation of the program and facilitators.

### Data management/analysis

At the conclusion of the intervention period, each fidelity checklist was entered into an Excel software program and a descriptive analysis combined the ratings for the five sessions, each evaluated six times. Field notes were typed verbatim into a Word document. Two of the authors used techniques of analyzing ethnographic field notes that included close reading, open coding, and the writing of initial memos. Significant words and phrases in the data that were relevant to fidelity were labeled and assigned preliminary codes by each investigator [14]. Areas of coding disagreement were discussed and resolved. Passages were grouped together and tentative category labels assigned to each group. To facilitate the detection of themes, data were organized into matrices of related content [15]. The remaining authors reviewed the analysis and offered opinions about the organization of the findings.

### Ethics/consent to participate

The Institutional Review Board of the University of Kansas Medical Center reviewed and provided approval to complete this project, number 13559: Sexual Health Empowerment for Cervical Health Literacy and Cancer Prevention. Due to the special population served by this study (incarcerated women) a Certificate of Confidentiality was also obtained through the National Institutes of Health to protect any identifiable research information. When introducing the study, women’s questions were answered and each woman signed an individual consent to participate form.

## Results

Each of the five sessions of the overall curriculum was evaluated six times (three for the intervention groups and three for the wait-listed comparison groups); institutional scheduling problems necessitated cancellation of five of the evaluation sessions. The mean score for adherence to the protocol content on the seven facilitator qualities were high, ranging from 8.8 to 9.9 (Table 1). Scores on the overall challenges encountered during the assessed session ranged from a low of 8 (minimal challenges) to 70 (many problems), with 2 days being over 50. The content elements included in each of the five sessions ranged from 77.3 to 88% (Table 2).

**Table 1:** Mean Ratings of Facilitator Quality during Five Sessions

Quality/Session	1	2	3	4	5	Mean Rating (SD)
Facilitating open-ended discussion	10	9	9.4	9.8	9.6	9.6 (0.3)
Expressing empathy	10	10	10	9.8	9.8	9.9 (0.1)
Engaging audience	9.4	9.2	9.4	9.3	9.4	9.3 (0.08)
Avoiding confrontation	9.6	9.8	9.8	9.8	9.6	9.7 (0.1)
Adhering to curriculum	9	9	8	8.8	9	8.8 (0.4)
Overall effectiveness of session	9	8.8	8.4	8.8	9.2	8.8 (0.3)
Adjusting to group’s pace/style	9.8	10	9.4	9.5	10	9.7 (0.2)
Overall challenge of group/ day (100% = most difficult)	20%	36%	52%	70%	80%	3.7 (2.2)

**Table 2:** Number of Content Elements Included in Observed Sessions

Assessment	#1	#2	#3	#4	#5	#6	Mean
Session 1	3/8	6/8	6/8	7/8	7/8	8/8	77.3%
	38%	75%	75%	88%	88%	100%	
Session 2	8/11	10/11	9/11	11/11	11/11	9/11	88%
	73%	91%	82%	100%	100%	82%	
Session 3	8/13	--*	11/13	10/13	10/13	--*	81%
	62%		85%	77%	77%		
Session 4	--*	--*	9/10	8/10	10/10	8/10	87.5%
			90%	80%	100%	80%	
Session 5	3/6	--*	6/6	6/6	6/6	4/6	83.4%
	50%		100%	100%	100%	67%	

*Program cancelled because of institutional issues

The qualitative data showed two categories of challenges to fidelity: one relating to the jail environment and a second relating to the participants’ response to content.

### Jail environment

The physical aspect of the jails was often not conducive to generating the type of conversation necessary to establish trust. For example, as excerpted from an evaluator’s field notes:

First time in this facility. The space is terrible in terms of seating and the visibility of the display board and in terms of acoustics. At about the midpoint of the session, the level of other activity in the room increased. Several staff members entered through the secured doorway to talk to the guard on duty. The door was operated by a motor of some kind and made a lot of noise opening and closing. At different points during the session, a male guard, a woman who looked like an administrator type, and two nurses entered. Our women were also up and moving around throughout the session—heating coffee in the microwave, getting water—all while our facilitator was talking. One woman disappeared during a bathroom break. We don’t know where she went or why she didn’t return. Two other women went to the desk and visited with the nurses in the middle of the session: one of them had her blood pressure checked. The other seemed to be having something on her foot examined.

In another facility:

The environment is a bit gloomy. The classroom has poor lighting and is small. The women must be escorted to and from the bathroom. A correctional officer is called when the session ends to transport women back to their cells. The women are concerned about being searched for pens and ask us to certify that we had collected the pens used. These factors impact the women’s sense of freedom and willingness to share.

The administrative operations of the jails resulted in frequent delayed starts and missed sessions as a result of lockdowns, staffing shortages, or miscommunication, all of which necessitated doubling up of programs and content. For example:

The women did not arrive until 9:25 am (scheduled for 9:00 am).

The session started at 9:31, per the clock in the room.

The session started late because there was an issue with the jail’s schedule sheet (we were not listed on it).

This week got scrunched together, because the Monday (Day 1) was MLK day. Jail staff would not let us do programming that day. Facilitators did a bit of Day 3 ending, and then got to Day 4.

No programming yesterday because of facility lockdown.

Session 1 did not start until the second day of the intervention. This means that session 3 and 4 were combined into one session resulting in abbreviation of curriculum.

*Participants* themselves were perceived by assessors as distractible, frequently diverging from session topics or showing distress over their own comments or those made by others. Allowing the women to be heard and acknowledged, while gently bringing the conversation back to the intervention topic, was often time-consuming, as illustrated in this field note:

We were unable to start session one due to the difficult scenario with the participants. In debriefing with the facilitators, the jail coordinator and I [the fidelity assessor] shared how this was the most difficult group of women yet experienced during this intervention. The jail coordinator mentioned that she almost called a “code” or program cancellation. One facilitator felt unsettled by some of the women’s comments towards her during the administration of the survey. I shared that it was a stressful 2h. I was overwhelmed by the difficulty of redirecting participants, the constant background chatter, and not knowing the best methods to diffuse the seeming chaos.

The group required redirecting on multiple occasions. Once redirected, the women were able to focus on the current topic.

Throughout the reading of the consent form there were many interjections. The facilitator remained calm while listening and answering questions, but also discreetly tried to draw the group back and keep them focused. Participant A continued to interrupt throughout entire session to share her thoughts and personal experiences. The facilitators were always extremely patient with her. The other women began to exchange looks of annoyance after the first few interruptions.

The women were exuberant/excited to be present. Several women preferred to talk amongst themselves which made for a distracting environment. The facilitator attempted to redirect the women on multiple occasions.

During the question and answer session Participants B and C openly agreed/disagreed with certain questions. Their comments were distracting and required redirecting by the facilitators.

There was an initial conflict between Participants D and E in the front of the room and third woman who was present, even though she had attended a previous program. After this third woman was removed, the remaining participants calmed down and were engaged.

The women were easily distracted today – they said so themselves.

A second challenge of the participants was related to the topics that were discussed and the need to allow time for emotional processing:

As the discussion began, women shared their experiences with STI’s and eventually rape. This caused emotions to rise. Participant F eventually started crying and expressed an inability to focus on the questions.

Participant G shared a long story about how drug use ruins your life and has caused her to lose her children as well as giving her endocarditis. She became very emotional and started crying. Once the women leave, the facilitators shared that this was a very emotional group and they were not able to make it through a majority of the material.

Participant H talked about fighting to get her kids back again. The discussion became very emotional.

Group was talkative and shared many personal stories and continually tried to offer advice to the other women. During these times, emotions tended to escalate and it was somewhat difficult to calm the group and regain focus.

The facilitator specifically mentioned to me before the session began that she would not be covering the women’s thoughts about healthcare in prison [an intervention topic], as their opinions were very clear from previous days and this topic is one that easily causes the women to get distracted and upset.

Due to the heaviness of the previous day’s discussion, some topics were missed so the facilitators took time to review and cover those remaining topics [today].

It appeared that two of the participants may have some form of mental illness based on their comments (tangential/circumferential) and difficulty staying on topic. Both women tend to become fixated on particular topics (rescuing women from prostitution homes and government conspiracy theories). Based on the difficult nature of two of the participants, it was difficult to appropriately evaluate fidelity for this session. The curriculum was altered by staff to be able to complete the 5-day intervention within a shortened period of time. The women had to be redirected on multiple occasions.

Several topics were not discussed due to participant difficulties (mental illness, tangential conversation, circumferential conversation, volatile environment).

Mentioned several times in the field notes was the critical role of the facilitators who adapted and varied delivery of intervention in order to:

- Negotiate with jail administration and correctional officers around schedules;
- Juggle content to address particular emotional needs of the participants;
- Tactfully (and repeatedly) control side conversations;
- Use humor, street-talk, and occasional examples from their own lives to make the material more comfortable and accessible;
- Avoid expressions of surprise, judgment, tension, doubt, or criticism when women described events of drug use, sexual behavior, abuse, or violence;
- Consistently indicate an awareness of the women’s incarcerated circumstances and the possibility that they may face constrained choices when not incarcerated.

## Discussion

Our finding of 77–88% adherence to protocol content in this criminal justice intervention is somewhat better than the findings of a similar program with incarcerated women that found 75% adherence, and in line with those found in interventions conducted in other community settings [16, 17]. For example, in their physical activity intervention delivered in preschool setting, Alhassan and Whitt-Glover (2014) found 67–68% of teachers delivered the program as instructed, citing time limitations as a major impediment [18]. The use of a video assessment tool in a community-based peer support trial on diabetes outcomes found only 42–44% adherence to the goal of nondirective counseling and the setting of specific goals [19]. A mental health intervention for war-affected Sierra Leonean youth found fidelity scores ranging from 2.89 to 3.87 out of a possible 4.00 [20]. The process evaluation of a county reentry program used four fidelity criteria: adherence, exposure, delivery quality and participation responsiveness. High ratings were found on the first three, with less positive scores found on adherence to program design. The authors attributed this last score to continuous administrative turnover and an unstable facility climate, conditions similar to those found with our implementation [17]. The best laid plans of community researchers must face and accommodate to real life participants and environments.

While community-based research necessitates attention to fidelity procedures that are very different from those seen in clinical research settings, interventions conducted in criminal justice settings pose additional challenges [21]. Our research staff spent extensive time in planning for the implementation of our intervention, including early, extensive discussion with jail administration, recognition of the diverse missions of jail staff and academic researchers, pilot testing of all program content, and acknowledgement of variations in the cultures of different facilities. These activities are supported by the academic literature [22, 23]. Such recommendations, however, do not provide guidance about the day-to-day challenges of facility cancellations and delays that were a constraint to protocol fidelity in our findings. The realities of conducting research in jails and prisons must be addressed in real time by juggling, prioritizing, or otherwise adjusting program content to fit the new timeframe. Likewise, ensuring that program content is engaging and appropriate through pilot testing and curriculum revision can assure material that is absorbing enough to move beyond the limitations of an unpleasant physical environment.

Our finding that the responses of participants to intervention content contributed to fidelity challenges should also be seen as an implementation strength. Women in the criminal justice system have a complex history of trauma, substance abuse, and mental health disorders. Program participation and group interactions was difficult for some. Intervention topics, especially those related to sexual health, often elicited painful responses. Researchers working with incarcerated women in Rhode Island acknowledged the reality that “trauma is inherent in corrections research” and the need for creative solutions in working with participants [24]. Behaviors that are the result of women’s trauma histories must be acknowledged and skillfully addressed to be able to continue with intervention content. To prioritize otherwise would be a disservice to the very population for which our intervention content is geared.

Limitations of the present study include the use of four assessors, whose perspectives may have introduced variation in the data collection.

## Conclusion

Researchers work with the goal of providing new knowledge that will result in improved health for target populations. Intervention fidelity with vulnerable groups must strike a delicate balance between protocol adherence and accommodation to participant needs. Our results suggest that skilled facilitators are critical for this effort. Additional attention to strategies that can strike this balance will benefit both participants and researchers.

## Abbreviations

HIV: Human Immunodeficiency Virus
IRB: Institutional Review Board
STI: Sexually Transmitted Infections
TB: Tuberculosis
